# Interpretive adaptability and siloization of quality in intensive care: a multi-level, qualitative analysis

**DOI:** 10.1108/JHOM-09-2025-0610

**Published:** 2026-04-28

**Authors:** Mia Hylén, Rebecca Selberg

**Affiliations:** Department of Intensive and Perioperative Care, Skane University Hospital, Malmo, Sweden; Department of Sociology, Lund University, Lund, Sweden; Department of Intensive and Perioperative Care, Skane University Hospital, Lund, Sweden

**Keywords:** Quality of care, Quality indicators, Intensive care, Collaboration, Siloization, Quality registries, Interpretive adaptability, Interprofessional reflexivity

## Abstract

**Purpose:**

Healthcare organizations increasingly rely on measurement practices to assess and improve care quality. However, how “quality” is conceptualized across different levels of the healthcare system remains unclear. This study explores perceptions of quality and quality indicators in intensive care, using two Swedish ICUs as case studies.

**Design/methodology/approach:**

The study examines two general ICUs in Sweden and is based on 16 semi-structured interviews with stakeholders at operational, clinical and national levels, complemented by document analysis. The documents include materials related to ICU operational development and national guidelines for knowledge management.

**Findings:**

The study identifies divergent – and at times conflicting – views on what constitutes relevant quality indicators in intensive care. It introduces two interrelated themes: interpretive adaptability, referring to the flexible meanings of quality depending on context, audience and institutional demands; and siloization of quality conceptualizations, describing how understandings of quality often evolve within professional and organizational silos, with limited cross-level communication or mutual awareness.

**Practical implications:**

The findings highlight the complexity of “quality” in healthcare and call for more reflexive and inclusive dialogues around quality assurance. A more collaborative and comprehensive approach may strengthen efforts to improve care.

**Originality/value:**

This study provides a unique empirical perspective on the varied meanings of quality in healthcare. It offers both theoretical and practical insights into the disconnect between formalized quality indicators and how key actors interpret and engage with the concept of quality.

## Introduction

What constitutes “quality” in healthcare remains a subject of ongoing debate, despite decades of efforts to define, measure and improve it. Across healthcare systems, quality is increasingly framed as something that must be “systematically and continuously developed and ensured” (SOSFS, 2011) – a sentiment echoed in international policy and practice (Endalamaw *et al.*, 2024). Central to this agenda is the belief that measurement enables improvement: “We can only be sure to improve what we can actually measure,” as a NHS report stated (Department of Health, 2008).

This logic has driven the proliferation of quality indicators, benchmarking tools and audit systems across healthcare, supported by advances in information technology and data analytics (Quentin *et al.*, 2019; Breyerbre *et al.*, 2019). Yet, as many scholars have noted, the expansion of measurement has not resolved the conceptual ambiguity surrounding “quality.” Definitions remain contested, and the boundaries between care quality and health system performance are often blurred (Busse *et al.*, 2019; Örnerheim, 2013). Despite widespread commitment to continuous quality improvement, comparisons across institutions and systems remain fraught with methodological and interpretive challenges (Breyerbre *et al.*, 2019; Powell *et al.*, 2003).

This study investigates these tensions by examining how “quality” is understood and enacted in Swedish intensive care. Drawing on interviews with stakeholders at operational, managerial and national levels, we explore how quality indicators are interpreted, negotiated and occasionally resisted in practice. Our focus is not only on what is measured, but on how meaning is constructed around the concept of quality in everyday clinical and administrative work.

We approach this through the lenses of institutional theory, sensemaking and practice theory, which together allow us to conceptualize quality as a contested and context-sensitive construct. This perspective bridges the gap between formalized quality indicators and the lived realities of healthcare professionals, while also highlighting barriers to shared understanding and coordinated quality improvement across the healthcare system.

Empirically, the study is based on two urban university hospitals in Sweden and their general intensive care units. Through interviews and document analysis, we uncover how different stakeholders – from frontline ICU managers to national registry officers – construct and apply divergent notions of quality. Our aim is twofold: to develop an empirically grounded understanding of how quality is perceived and practiced across levels of the healthcare system and to offer conceptual tools for fostering more reflexive and inclusive approaches to quality improvement.

We begin by situating the concept of quality within healthcare and specifically within the Swedish healthcare system, followed by a description of our methods and research sites. The results section then examines how quality is interpreted and enacted in intensive care, highlighting the tensions and adaptations that emerge in everyday clinical practice.

## Background: quality in the healthcare system

When assessing hospital performance internationally, there is often an overemphasis on quantitative indicators such as efficiency, productivity, safety and effectiveness (Hadian *et al.*, 2024), all of which are readily measurable. The more abstract concept of quality is frequently overlooked in such evaluations. As a result, quality tends to be highly context dependent and is often defined from the perspective of the user (Harteloh, 2003). A concept analysis of quality in healthcare proposes that it entails “the assessment and provision of effective and safe care, reflected in a culture of excellence, resulting in the attainment of optimal or desired health”, thereby highlighting its foundational components. What is considered “optimal” or “desired” is ultimately shaped by the recipient of care – the patient (Allen-Duck *et al.*, 2017).

The concept of “quality” has become central to healthcare governance in Sweden, shaped by decades of reform influenced by New Public Management (NPM) and global trends in performance-based management. Since the late 1980s, Swedish public sector institutions have increasingly adopted managerial frameworks such as total quality management and ISO standardization, emphasizing efficiency, outputs and quantifiable performance (Örnerheim, 2013; Modell and Grönlund, 2007). These developments have led to the proliferation of quality indicators and measurement systems across healthcare, culminating in the establishment of national quality registries.

These registries – now coordinated under the National System for Knowledge-Driven Management (NSKM) – are designed to support evidence-based care by collecting detailed, person-based data on treatments and outcomes. The NSKM's motto, “We count our success in lives and equal health,” reflects a foundational commitment to measurement and data-driven decision-making. Yet, this emphasis on quantification exists within a healthcare system marked by structural fragmentation: responsibilities are divided among the national government, 21 regional authorities and 290 municipalities, each with independent taxation powers and varying degrees of reliance on private providers (Svallfors and Tyllström, 2019).

Despite its sophisticated data infrastructure, the Swedish healthcare system faces persistent challenges, including staff shortages, long wait times and regional disparities in care (Falkenström and Svallfors, 2022). These tensions highlight a paradox: while Sweden performs well on international quality benchmarks (OECD, 2024), domestic actors often experience the system as strained and uneven, with serious quality deficiencies.

The National Board of Health and Welfare defines six dimensions of quality – Swedish healthcare should be evidence-based, safe, individualized, efficient, equitable and accessible – but these dimensions remain broad and open to interpretation. As early as the 1990s, healthcare professionals criticized the vagueness of the quality concept, noting that it conceals competing logics: medical, managerial, experiential and ethical (Calltorp, 1996). These tensions persist today, as quality is invoked across multiple levels of the system – by clinicians, managers and policymakers – each with distinct priorities and epistemologies.

To understand how “quality” is enacted within this complex system, we draw on insights from institutional theory and practice-based approaches to organizational life. Institutions such as the NSKM provide formalized structures, rules and indicators that shape how quality is defined and measured. However, these structures do not determine practice in a linear way. Instead, actors interpret, negotiate and sometimes resist institutional logics in their everyday work (Thornton *et al.*, 2012).

We also build on sensemaking theory (Weick, 1995), which emphasizes how individuals construct meaning in complex and ambiguous environments. In the context of intensive care – where outcomes are uncertain and ethical stakes are high – professionals must continuously interpret what “quality” means in relation to specific patients, situations and institutional expectations.

Finally, we are informed by practice theory (Gherardi, 2012), which highlights how knowledge and meaning are produced through situated actions. From this perspective, quality is not only a measurable outcome but also a social accomplishment – something enacted through interactions, judgments and negotiations among professionals, patients and systems.

Together, these perspectives allow us to conceptualize quality not as a fixed or universally agreed-upon standard, but as a contested and context-sensitive construct. This framing enables us to explore how structural frameworks (such as quality registries and national guidelines) are interpreted and enacted by actors at different levels of the healthcare system. The principal objective is to explore perceptions of quality and quality indicators in intensive care, using two Swedish ICUs as case studies.

## Methods and research site

### Research design and sites

This study employed a qualitative research design, combining semi-structured interviews with document analysis. This approach is well-suited for exploring meaning-making and experiential knowledge in complex organizational settings (Patton, 2014; Leavy, 2024). The research was conducted in two general intensive care units (ICUs) at a Swedish university hospital, representing the highest level of intensive care nationally.

ICUs are data-intensive environments where advanced monitoring technologies intersect with continuous human observation. They are characterized by both “high-tech” and “high-touch” care, where clinical decisions rely not only on measurable indicators but also on sensory and relational knowledge. These settings offer a rich context for examining how quality is conceptualized and enacted.

### Participants and data collection

Data were collected through 11 individual interviews and two focus groups (*n* = 3, *n* = 2) with key stakeholders, including clinical and administrative managers and individuals responsible for quality data at both local and national levels. Participants were selected based on their roles in quality assessment and improvement, and all had extensive experience in their respective positions.

Interviews lasted 45–60 min, were conducted either in person or digitally and were audio-recorded with informed consent. A semi-structured interview guide focused on how quality is measured, interpreted and used in decision-making. All interviews were transcribed verbatim. To enhance trustworthiness, some interviews were conducted jointly by the authors at different stages of data collection.

In addition to interviews, we analyzed national and local documents related to quality management in intensive care, including policy guidelines, benchmarking reports and internal audit materials from the national quality registry for intensive care (SIR). These documents were selected to reflect both regulatory frameworks and operational practices (see Table 1).

**Table 1:** Analyzed national and local documents related to quality management in intensive care

Designation	Name of document; author/issued by; year/date	Translated title and type of document	Internet link (accessed)
D1	Varför jämföra oss när vi vet bäst? Svensk hälso- och sjukvård i ett jämförande perspektiv. Peter Daneryd, Forum för Health Policy (2015)	Why compare when we know best? Swedish healthcare in a comparative perspective *Report published in collaboration with the trade association for the research-based pharmaceutical industry in Sweden*	https://healthpolicy.se/wp-content/uploads/2021/11/Daneryd_Sv_vrd_intl_jfr.pdf (2025–08-21)
D2	Kvalitetsindika-torer för svensk intensivvård. Per Lindgren, Thomas Nolin, Johan Petersson Svenska Intensivvårds-registret (SIR, 13–03-15)	Quality Indicators for Swedish Intensive Care *Training course presentation material, Swedish Intensive Care Registry*	https://www.icuregswe.org/globalassets/moten/vargard2013/kvalitetsindikatorer_saltsjobaden_2013.pdf (2025–08-21)
D3	SIR Audit 2019 Östersund	Swedish Intensive Care Registry Audit for Östersund hospital *Audit report from the Swedish Intensive Care Registry*	https://www.icuregswe.org/forbattringstorg/audit-tips-och-dokument/(2025–08-21)
D4	SIR Audit 2019 Uppsala CIVA	Swedish Intensive Care Registry Audit for Uppsala hospital *Audit report from the Swedish Intensive Care Registry*	https://www.icuregswe.org/forbattringstorg/audit-tips-och-dokument/(2025–08-21)
D5	SIR Audit 2021 Huddinge	Swedish Intensive Care Registry Audit for Huddinge hospital *Audit report from the Swedish Intensive Care Registry*	https://www.icuregswe.org/forbattringstorg/audit-tips-och-dokument/(2025–08-21)
D6	SIR Audit 2021 Södertälje	Swedish Intensive Care Registry Audit for Södertälje hospital *Audit report from the Swedish Intensive Care Registry*	https://www.icuregswe.org/forbattringstorg/audit-tips-och-dokument/(2025–08-21)
D7	SIR Audit 2021 Kalmar	Swedish Intensive Care Registry Audit for Kalmar hospital *Audit report from the Swedish Intensive Care Registry*	https://www.icuregswe.org/forbattringstorg/audit-tips-och-dokument/(2025–08-21)
D8	SIR Audit 2022 Sunderby	Swedish Intensive Care Registry Audit for Kalmar hospital *Audit report from the Swedish Intensive Care Registry*	https://www.icuregswe.org/forbattringstorg/audit-tips-och-dokument/(2025–08-21)
D9	SIR Audit 2022 Eksjö	Swedish Intensive Care Registry Audit for Eksjö hospital *Audit report from the Swedish Intensive Care Registry*	https://www.icuregswe.org/forbattringstorg/audit-tips-och-dokument/(2025–08-21)
D10	SIR Audit 2022 Norrköping	Swedish Intensive Care Registry Audit for Norrköping hospital *Audit report from the Swedish Intensive Care Registry*	https://www.icuregswe.org/forbattringstorg/audit-tips-och-dokument/(2025–08-21)
D11	SIR Audit 2023 Borås	Swedish Intensive Care Registry Audit for Borås hospital *Audit report from the Swedish Intensive Care Registry*	https://www.icuregswe.org/forbattringstorg/audit-tips-och-dokument/(2025–08-21)
D12	SIR Audit 2023 Falun	Swedish Intensive Care Registry Audit for Falun hospital *Audit report from the Swedish Intensive Care Registry*	https://www.icuregswe.org/forbattringstorg/audit-tips-och-dokument/(2025–08-21)
D13	SIR Audit 2023 Umeå	Swedish Intensive Care Registry Audit for Umeå hospital *Audit report from the Swedish Intensive Care Registry*	https://www.icuregswe.org/forbattringstorg/audit-tips-och-dokument/(2025–08-21)
D14	SIR Audit Säker kvalitet intensivvård 2022	Presentation material, Swedish Intensive Care Registry audit 2022	https://www.icuregswe.org/forbattringstorg/infor-kommande-audit/(2025–08-21)
D15	Verksamhets-dialog våren 2021 (anonymized)	Yearly operations dialogue template 2021 (anonymized)	Not available for dissemination
D16	Verksamhets-dialog hösten 2022 (anonymized)	Yearly operations dialogue template fall 2022 (anonymized)	Not available for dissemination
D17	Verksamhets-dialog hösten 2023 (anonymized)	Yearly operations dialogue template fall 2023 (anonymized)	Not available for dissemination
D18	Presentations-material, sjukhus-förvaltningens ledningsgrupp: kvalitets-uppföljning (anonymized)	Presentation material for the hospital management group on quality assurance (anonymized)	Not available for dissemination

**Source(s):** Authors’ own work

### Data analysis

Interview and document data were analyzed using an inductive, reflexive approach. Transcripts were coded both descriptively and analytically, with codes linked to emerging themes and theoretical concepts (Deterding and Waters, 2021). Coding was conducted independently by the authors, then compared and refined through collaborative discussion. Documents were read iteratively and coded to identify how quality was defined, operationalized and enacted across institutional levels (Bowen, 2009; Prior, 2008). This triangulation of sources enabled a deeper understanding of the discursive and practical frameworks shaping quality work in intensive care.

### Ethical considerations and anonymity

The study was approved by the Swedish Ethical Review Authority (Dnr, 2023–01992-01) and by operational managers at both research sites. Participants received detailed information about the study and provided informed oral consent. To protect participants' identities – particularly given their identifiable roles in specific organizational contexts – we chose not to include a detailed participant table. While this limits transparency, we prioritized confidentiality to ensure participants could speak freely about sensitive organizational issues.

## Results

Interviews revealed a diverse yet consistently nuanced understanding of quality in care. All participants described challenges in approaching “quality” as a generalized concept encompassing all aspects of care provision and development. They also reported using the concept in varied ways, referring to different practices, standards and expectations depending on context and interlocutor. As one ICU manager remarked when responding to the interview request: *“The question* [about the meaning of quality] *is interesting and complex – anyone who says there is a simple answer is either lying or ignorant*.” [1] This statement reflects a shared awareness of the multifaceted nature of quality in intensive care.

The rich empirical material generated through interviews and document analysis revealed two overarching themes: Interpretive adaptability and diverse meanings of quality – how staff navigate multiple, context-dependent understandings of quality. Siloization of quality conceptualizations **–** the limited reflection and dialogue about quality across organizational levels and professional boundaries.

### Theme 1: Interpretive adaptability and diverse meanings of quality

For ICU staff, “quality” was not a fixed or easily defined concept. Most interviewees struggled to offer a general definition, instead describing quality in relation to specific situations, goals and patient needs. As one clinician explained: “*Quality can be so many different things … it's totally dependent on who the patient is.”*

In some cases, high-quality intensive care meant helping a patient recover from organ failure; in others, it meant ensuring a peaceful and dignified death. These definitions reflect a deeply patient-centered view of quality, grounded in clinical experience rather than static indicators.

### Shifting between frameworks

While clinicians emphasized patient-centered care, they also acknowledged other definitions of quality – particularly those tied to national indicators. Many moved fluidly between different frameworks. For example, interviewees might cite mortality rates or readmission statistics, then shift to discussing communication with families or emotional outcomes as alternative markers of quality. This ability to adjust their understanding depending on context is what we refer to as *interpretive adaptability*.

One nurse manager reflected on this tension:

You can have great survival rates, but that doesn’t necessarily mean it’s been good care.

Another added, arguing that a good outcome can be peaceful death and a family that feels supported:

Obviously, it would not be good intensive care if patients died at a higher-than-expected rate […] But, good intensive care can also be those instances where it didn’t end well for the patient, but there was a well-functioning cooperation between kin and staff. And you can’t measure that. One of the strongest memories I have is of a young woman who died. And her mother said when she left the ward, “it was good.” And I thought, yes, it was a good ending, under the circumstances. It didn’t look good in our statistics, but it was a good ending—and how you measure that, I don’t know.

This reflection captures a central tension: while statistical indicators like mortality rates are necessary for reporting and benchmarking, they often fail to reflect the emotional, ethical and relational dimensions of care. For the interviewed clinicians, quality is not just about survival – it is about how care is experienced by patients and families. Their ability to move between these different understandings illustrates the interpretive adaptability required to navigate the complex realities of intensive care.

### Limits of indicators

Mortality rate was frequently cited as an example of the limitations of static indicators. One clinician noted that while it is a key metric, it can be misleading or manipulated. Referring to a neighboring unit, the interviewee joked: *“They tend to keep their patients for 31 days, and their 30-day mortality rate is top-notch, naturally.” “Keeping patients alive is not the problem”*, this interviewee explained, referring to the advanced life-supporting machinery available to all ICUs; the challenging part of intensive care medicine is *“knowing how far to push the care”* in service of the patient, and making *“the right decisions at the right time in each unique case”*. This, the interviewee said, is *“not easy to measure or evaluate”* and *“cannot be readily gleaned from statistics”*. The contrast between low mortality rate as evidence of successful intensive care and a good outcome in terms of, for example, a peaceful and dignified death, captures the width and complexity of the quality concept in the interviewees' narratives.

Others pointed out that registry data often failed to capture the complexity of ICU cases, especially when patients arrived in critical condition but improved rapidly. A physician explained [2]:

*T*he [quality registry] is good, but what you get out of it is what you put into it. […] Based on vitals you get a predicted mortality … and then you look for the observed mortality compared to this predicted mortality. Are we better off or worse off? If we have a higher than predicted mortality rate, then we’ll get a standardized value above one. If we’re below, it’s below one. The problem is that these numbers are registered about an hour after the patient arrives. So, you have a patient coming to the emergency department in cardiac arrest. Zero blood pressure, zero pulse, super bad parameters and high risk of death. And then it goes to the cath lab and ninety minutes later he arrives in the ICU with a normal pulse and normal pressure. Ok, but he was close to death initially – and the registry can’t catch that. […] We’ll get a false low mortality expectancy. So, all of this you need to include when you look at your quality parameters – what do they mean, really? … Let’s say we were at a two for mortality rate, “How can you register at a two, are you killing all your patients?” I’d need to have an answer on that. But most likely it’ll be a register technicality. That is why I try to follow up on our patients regularly. What patients do we have? What are we doing with them? To me, that is a more important quality assessment.

Despite these limitations, quality registries were not dismissed. Staff used them to identify trends, communicate with leadership and validate their own experiences. For example, registry data confirmed staff suspicions that overdose cases had increased after the pandemic or that pediatric admissions rose during summer. These moments of confirmation were described as morale-boosting, helping staff feel seen and valued. As one ICU manager noted:

The only time I take out data from the quality registry is to present it to the staff, on yearly staff days. They like hearing about it—how many patients they’ve treated and so on. It’s more of a morale-boosting exercise. They’d be disappointed if I didn’t present any of it. It plays the role of showing them how much they’ve accomplished in a year, I suppose.

Despite the presence of formal indicators, most interviewees emphasized that meaningful quality assessment occurred through direct engagement. As one participant explained:

It’s much better to be present, to talk to colleagues, to follow up on patients. That’s how you know where we are in terms of quality.

This sentiment reflects a preference for relational and situated forms of evaluation, grounded in clinical judgment and peer dialogue rather than abstract metrics.

### Quality in national documents

National policy documents and registry frameworks typically define quality in terms of compliance, documentation, and measurable outcomes. These frameworks often follow Donabedian's (1988) model of structure, process and outcome indicators. For example, one report (D1) on Swedish healthcare in a comparative perspective states:

The large databases of the Swedish healthcare system offer strong potential for turning Big Data into Fast Data, which is a prerequisite for using healthcare outcomes in evidence-based decision-making to develop, lead, and manage in real time (D1, p. 7).

This vision contrasts sharply with the practices described by ICU clinicians, who rarely use Big Data or Fast Data to guide real-time decision-making. Instead, their quality work is embedded in clinical routines, interpersonal communication, and reflective practice.

While national frameworks aim to support both local improvement and system-wide oversight, they also acknowledge their own limitations – particularly the need for contextual interpretation. Documents from the Swedish Intensive Care Registry (SIR) reflect this duality. While promoting structured indicators, they also encourage reflective use of data. For instance, in a presentation on quality indicators (D2, p. 25) SIR notes that hospital management and national authorities link “the use of quality indicators to economic reimbursement,” and warns of “negative effects of national quality indicators,” including: “a narrowed focus on quality and quality work” that risk “undermining locally adapted focus on quality” (D2, p. 28). Local protocols and audit tools collected by SIR (D3–D14) are consequently framed not only as compliance mechanisms, but as part of a learning culture.

Across interviews and documents, a clear pattern emerged: ICU professionals do not reject formal indicators, but they do not rely on them exclusively or even extensively. Instead, clinicians navigate between institutional demands and professional judgment. Quality is not simply measured – it is interpreted, enacted and negotiated in practice. These findings illustrate how institutional logics – embodied in national guidelines, quality registries and managerial frameworks – provide formal structures for defining and measuring quality. However, as institutional theory suggests, these structures are not determinative; rather, they are interpreted and adapted by actors within specific local contexts, leading to diverse enactments of “quality” in practice (see, e.g. Vivier *et al.*, 2024). The observed interpretive adaptability among ICU staff further reflects ongoing sensemaking processes, where individuals and teams construct meaning around “quality” in response to ambiguous, complex and shifting clinical realities. Recent sensemaking research emphasizes that such interpretations are shaped by interactions, experiences and the need to reconcile multiple, sometimes competing, expectations (Sandberg and Tsoukas, 2020). From a practice theory perspective, the enactment of quality is not merely a matter of applying abstract standards, but is accomplished through situated actions, judgments and negotiations in everyday clinical work. Quality thus emerges as a practical accomplishment, embedded in the routines and embodied knowledge of healthcare professionals (Endalamaw *et al.*, 2024).

This *interpretive adaptability*, then, enables staff to meet both bureaucratic and clinical expectations. However, it also reveals a deeper issue: the fragmentation of quality discourses across organizational levels. This fragmentation – and the lack of shared understanding between roles and professions – is the focus of the next section.

### Theme 2: The siloization of quality conceptualization

While participants across professional roles demonstrated notable interpretive adaptability in how they navigated and enacted quality in intensive care, our analysis revealed a striking lack of inter-professional dialogue or shared understanding of what quality entails. Physicians and ICU specialist nurses consistently emphasized different aspects of quality, often grounded in their respective clinical logics and professional responsibilities.

As previously discussed, physicians did not primarily focus on measurable outcomes and biomedical indicators, as might be expected. Instead, they reflected on issues such as dignity, communication and quality of life when addressing the complexities of defining “quality” in intensive care. However, interviewed nurses often assumed that physicians viewed quality through a biomedical and quantitative lens. Meanwhile, physicians struggled to imagine nurses' approaches to quality from the distinct perspective of the nursing profession.

These divergent conceptualizations were not necessarily in conflict, but they operated in parallel – rarely intersecting in ways that fostered mutual understanding or collaborative development of quality indicators. This dynamic is illustrated in the following exchange with a first-line physician manager:

**Interviewer:** Do you think there is an inter-professional discussion around [quality indicators], about what the most important indicators are, for example?

**ICU Physician:** [Silence]

**Interviewer:** So, between nurses and physicians, maybe nursing assistants, the para-medical professions? Is there a discussion there?

**ICU Physician:** I don’t fully understand the question.

**Interviewer:** Right, let me put it this way. If I were to ask a nurse here [at the ICU]: “What are the most important quality indicators, what do you need to look for to know if you’re providing high-quality care”—would she or he say the same thing as you?

**ICU Physician:** I think it would be more about pushing the care too far. They can sometimes feel like we push it too far. It feels unethical.

**Interviewer:** Do you use the same quality indicators, physicians and nurses?

**ICU Physician:** That’s an interesting question.

In contrast, discussions with nurses and nurse managers centered on the absence of meaningful measurement practices for assessing the quality of nursing care. The following excerpts from two focus group interviews – one with three first-line managers (M) and another with two follow-up clinic managers (FM) – illustrate this point:

**M1:** We don’t measure much at all, actually.

**M3:** Pressure wounds is something we look at.

**M2:** Hygiene, following hygiene protocols.

**M1:** Absolutely. Delirium.

**M3:** But we don’t have a good measurement of [delirium].

**M1:** No, I’m saying, we could …

**M2:** You’d think, since delirium is associated with mortality.

**M3:** Pain—we have a group working on pain issues, we could do that more …

**FM1:** In [the quality registry], I think there are no good quality indicators for nursing care. […] What I think is good care, if you can give that, it is also this thing where you enter an ICU and there’s calm. […] If there is someone bedside all the time, someone present. I don’t know, I imagine the care is better then. Patients aren’t alone in the room, and then we can decrease sedation and have a more awake patient. It’s difficult to measure that.

**FM2:** And things like, has the patient been sitting up today? Have they been offered that possibility? Or what stopped the patient from being mobilized?

**FM1:** Or did we have time to write in the patient diary? […] If we could evaluate that, these care bits …

**FM2:** And delirium, too. What did you do to reorient the patient? That is something we could measure.

**FM1:** Yes, we could measure that. That’s what I’d like to see measured—these care efforts. Because precisely this thing about quality—it’s so easy to measure the tangible stuff … But these things that we actually do, wash someone’s hair or shower them. If we took a patient for a walk in the building. That should be [noted].

**FM2:** How quickly do we wash off a trauma patient all this blood? Or are they left lying there looking dirty from the blood?

These extensive excerpts illustrate, first, the difficulty physicians experienced in imagining which practices and outcomes might constitute relevant quality indicators in nursing care. This reflects the dominance of the medical profession: nurses were well aware of the key indicators used to evaluate medical care – such as risk-adjusted mortality rates and readmissions – while physicians could afford to remain unaware of nursing-specific quality indicators.

Second, the excerpts highlight nurses' shared awareness that the core of nursing care – its relational, embodied and often subtle dimensions – is difficult to capture through existing measurement systems. Yet rather than rejecting measurement altogether, participants expressed a desire for more meaningful ways to evaluate nursing work. Their reflections point to a paradox: while nursing care is deeply contextual and often invisible in formal metrics, it is precisely this “invisible work” that they wish could be made visible – not only to validate their contributions but also to improve care quality.

The examples they offered – reorienting a delirious patient, ensuring mobilization, writing in the patient diary or simply maintaining a calm and present atmosphere – are not easily reduced to numerical indicators. Still, participants suggested that these aspects could be documented, tracked and reflected upon in ways that support both learning and accountability. In this sense, their comments reflect a form of *aspirational measurability*: a hope that the quality of nursing care might one day be evaluated on its own terms, rather than being overshadowed by more easily quantifiable medical outcomes.

### Quality or production?

The fragmentation – what we term the *siloization* of quality conceptualizations – was not merely a matter of differing professional perspectives but reflected deeper institutionalized separation. There was little evidence of curiosity about how other professional groups defined or assessed quality, and no structured forums for such exchange. Moreover, this professional siloization was mirrored – and arguably reinforced – by the organizational hierarchy.

From the vantage point of hospital management, quality indicators – carefully implemented through quality registries and the broader NSKM system and upheld by national frameworks – appeared largely irrelevant. What mattered was *production*: quantifiable throughput, occupancy rates and budget adherence. This focus was evident in documents from the annual “operations dialogues,” where clinic management reported to hospital leadership. These dialogues were structured around requests for specific performance data (D15, D16).

This managerial disinterest in quality as a clinical, organizational, or ethical concern further entrenched the disconnect between frontline professionals and upper-level decision-makers. When asked what hospital management wanted to know about quality in their operations, clinic managers consistently responded: *essentially nothing*. For example, two follow-up clinic managers noted:

**Interviewer:** Are you ever asked to share your insights with those higher up [in hospital management]?

**FM1:** Surprisingly rarely, considering how long we’ve been at it.

**FM2:** I think you’re the first one who’s ever asked us. So, no, I reckon we’re just a costly little half-complicating side business.

**FM1:** Our experience is that it’s rare [that anyone asks]. Very rare. Which is unfortunate, because there is so much to learn from [the follow-up clinic]. And it’s even stranger considering the county’s policies on patient satisfaction and all this. I’m like, well, ask us!

Similar sentiments were expressed by two physician managers:

**Interviewer:** Do you ever report numbers upwards?

**ICU Physician:** Yes, it happens.

**Interviewer:** What do they ask for?

**ICU Physician:** Production. How many inpatient days?

**Interviewer:** What type of data do you report upwards?

**ICU Physician:** Nothing.

**Interviewer:** They don’t ask you for data on reintubations or readmissions?

**ICU Physician:** No. I’ve been asked about occupancy rate, the number of admitted patients—stuff like that.

Another clinician summarized the situation bluntly:

From above, nothing is required [in terms of data on quality of care]. No, I don’t think they know anything about quality. I don’t think they care.

An operations developer at the hospital management level offered insight into how “quality” and “performance” were conflated. In discussing quality assurance work, the interviewee explained that hospital management had long focused on setting “overarching goals and strategies” that could then be properly evaluated, but acknowledged difficulties in communicating these goals to staff:

[Staff is not aware of the goals], you’re always surprised that people don’t seem to know why they’re at work. They should know. But most feel they don’t know. Or at least what the goals are, with what we are doing here.

**Interviewer:** Right, so you have these goals, one of which is highest medical results. How do you work with assessing how close you are to reaching the goals then?

**OD:** Well, we measure, accessibility for example, it’s measured nationally, or regionally, each month numbers are distributed. Each clinic works with this. [There are] production plans broken down on daily targets. […] But we are not close to reaching our goals, because we’re not at full capacity. For medical quality, we look at the national indicators. Hospital-acquired infections, bedsores. Anyway, we look at those national indicators where we know we’ll be followed up [by other authorities]. And then there are also more specialty-specific measures, for example how quickly a hip fracture is operated on after arriving at the emergency department, or how quickly someone receives treatment to dissolve a stroke after arriving with one, or how fast a heart attack is treated. There are certain indicators that are specific to areas of care, but which are still of general interest when assessing how well a hospital is functioning.

These findings highlight a critical paradox: while healthcare professionals demonstrate flexibility in how they interpret and apply the concept of quality within their own domains, there is little shared dialogue across professional or organizational boundaries. The gap is wide enough for hospital administrators to assume that clinicians do not know why they are at work or what the goals of their efforts are – an understanding that starkly contrasts with clinicians' nuanced and reflective approaches to quality.

In this context, quality becomes a fragmented and stratified construct, shaped by distinct logics, responsibilities and expectations – yet rarely discussed collectively. The result is a system in which quality is interpreted and enacted within silos: adaptively, but in isolation.

Indeed, between the operational and managerial levels, our findings suggest a conflation of the concepts of *quality* and *performance*. What is formally labeled as “quality assurance work” often centers not on clinical or ethical dimensions of care, but on metrics tied to quantitative output – such as patient throughput, bed occupancy and budget adherence. Clinicians themselves frequently referred to this as a focus on “production,” highlighting the disconnect between the logics of care and the logics of control.

The siloization of quality conceptualizations can be understood as a product of institutionalized boundaries between professions and organizational levels. Institutional theory highlights how distinct logics and routines become embedded within professional groups, reinforcing separation and limiting opportunities for shared understanding or integrated quality development (cf. Bode *et al.*, 2017). Sensemaking processes are often confined within professional silos, as clinicians and managers draw on their own experiences, values and peer interactions to interpret what quality means. The lack of interprofessional dialogue restricts the emergence of shared narratives, resulting in parallel but uncoordinated approaches to quality improvement. Practice theory draws attention to how knowledge and meaning are produced and sustained within specific communities of practice. The observed siloization reflects how quality work is enacted and reproduced within these communities, with limited mechanisms for cross-boundary learning or reflexivity. This limits opportunities for meaningful dialogue across levels and reinforces a fragmented system in which insights from frontline practice are rarely integrated into strategic decision-making. Without mechanisms for shared reflection, the system risks perpetuating isolated practices and missing opportunities for collaborative quality development.

## Discussion

This study set out to explore how quality is conceptualized and enacted in Swedish intensive care, revealing a landscape shaped by both interpretive adaptability and siloization. While clinicians demonstrated the ability to shift between different understandings of quality depending on context, these interpretations largely remained confined within professional and organizational boundaries.

To interpret these findings, we draw on institutional theory, sensemaking theory and practice-based approaches to organizational life. From an institutional perspective, the Swedish healthcare system is structured by overlapping logics – clinical, managerial and regulatory – that shape how quality is defined, measured and operationalized. Institutional theory helps explain how formal structures such as national quality registries and performance metrics become embedded in organizational routines through normative and regulative pressures (Jensen *et al.*, 2009). However, these structures are not passively adopted; they are interpreted and enacted by actors within specific contexts.

This is where sensemaking theory becomes essential. Our findings show that ICU professionals continuously interpret what quality means in relation to their roles, patients and institutional expectations. These interpretations are dynamic, evolving through interaction, reflection and experience (Weick *et al.*, 2005). Yet, this sensemaking largely occurs within silos. Physicians and nurses often operate with parallel but unspoken assumptions about quality, and there is little structured dialogue to bridge these perspectives.

This aligns with practice theory, which emphasizes that knowledge is produced through situated action and that organizational life is constituted through everyday practices (Gherardi, 2012). In our case, the practice of quality work is deeply contextual and embodied yet rarely shared across professional or hierarchical boundaries. While ICU teams are often praised for their interprofessional collaboration in acute situations (Lin *et al.*, 2022), our findings suggest that this collaboration does not extend to shared conceptual work around quality. Instead, discussions about quality – when they occur – tend to happen within homogenous professional groups.

Table 2 illustrates the discussed silos across different organizational levels – national, managerial and operational – demonstrating how each level relies on distinct bases of assessment, produces different outputs and faces unique barriers. These findings resonate with recent work on health system improvement, which emphasizes the need for interprofessional collaboration and reflexive dialogue to bridge the gap between system-level metrics and frontline realities (Eljiz *et al.*, 2023). Without such dialogue, the system risks reinforcing fragmented practices, where meaningful insights remain isolated and opportunities for integrated improvement are lost.

**Table 2:** Fragmentation of quality conceptualizations across organizational levels, an overview

Organizational level	Basis of assessment	Output	Consequences	Barriers- challengers
National	Quality registries	Standardization of quality indicators	No comparisons between hospital and units	Impenetrable, increased administrative burden
Managerial	Production (access, available beds, patient flows)	Budget and control	No reflection on quality	Stratification of knowledge bases between levels
Operational	Assessments, impressions of operations, experiences, knowledge of operation	Collective knowledge of quality of operations	No reflection or consensus on quality indicators	Silos between professions and functions

**Source(s):** Authors’ own work

The differences across organizational levels are particularly evident in the conflation of “quality” with “performance” at the managerial level, reflecting a broader institutional drift toward audit cultures and metric-driven governance. While these tools aim to support accountability, they often obscure the relational and ethical dimensions of care that professionals value most.

National quality registries are not reliable for benchmarking indicators across actors, and statements from registry representatives suggest a growing awareness of this limitation. Encouraging a more reflective use of data, they advocate for a learning culture in which data are not treated as static or absolute, but rather locally interpreted and discussed for improvement (D2). This may indicate an emerging recognition of the complexity of quality and a shift toward a more reflexive approach at the managerial level.

As our findings show, clinicians – particularly nurses – are not opposed to measurement *per se*. Rather, they express a desire for new forms of measurement that can capture the relational, embodied and often invisible aspects of care; they resist the binary conceptualization of “quantifiable” versus “non-measurable” practices and values that has surfaced in discussions around care professions in Sweden and internationally in recent years (cf. Ajana, 2018; Bornemark, 2018). This reflects what we term *aspirational measurability*: a hope that the quality of nursing care might one day be evaluated on its own terms, rather than being overshadowed by more straightforwardly quantifiable medical outcomes.

This siloization of quality conceptualizations is not only professional but institutional. Nurses were acutely aware of the indicators used to evaluate medical care, while physicians often struggled to articulate what quality meant from a nursing perspective. Meanwhile, hospital management appeared largely disengaged from clinical understandings of quality, focusing instead on performance metrics such as occupancy rates and throughput. This disconnect reinforces a fragmented system in which quality is measured, enacted and valued differently across levels – without mechanisms for integration or shared reflection.

We argue that this fragmentation reflects a broader lack of interprofessional reflexivity – that is, the structured, collective examination of how different professional groups understand and enact key concepts like quality. As McHugh *et al*. (2020) note, reflexivity in healthcare teams often centers on performance in specific situations, rather than on deeper conceptual or ethical reflection. Yet such reflection is essential if healthcare teams are to move beyond parallel logics and toward a more integrated understanding of care. As Baerheim *et al.* (2023) put it: “If every team member thought in the same way, there would be limited learning; it is in the interprofessional tension where most learning occurs.”

## Conclusion

This study offers a unique exploration of how quality and quality indicators are perceived within intensive care – one of the most data-driven domains in healthcare. The findings reveal a notable adaptability among clinicians in navigating diverse meanings of quality. They demonstrated awareness of multiple perspectives and applied them contextually. For instance, while formal indicators such as mortality rates were acknowledged as blunt instruments, they were still considered useful in certain situations. However, clinicians emphasized that quality should primarily be assessed from the patient's perspective, highlighting the principles of beneficence and non-maleficence as more meaningful.

Furthermore, the study uncovered both professional and organizational siloization in the conceptualization of quality. Discussions about the meaning of quality were rare across professional boundaries, limiting mutual understanding and hindering collaborative development in care quality. While medical, quantifiable outcomes were broadly accepted, nurses expressed an aspiration for the measurability of nursing care – a hope that its quality might one day be recognized, measured and evaluated on its own terms. Siloization was also evident across organizational levels. At higher levels, quality was often conflated with production or performance metrics, which were misaligned with the clinicians' more nuanced and patient-centered understanding of quality. This disconnect underscores the need for mechanisms that support integration and shared reflection across both professional and organizational boundaries.

## Implications for practice

The core implication of this study is the need for interprofessional reflexivity – structured reflection across professional groups about the meaning and assessment of quality. The diversity of perspectives in intensive care should not be viewed as a problem to be resolved, but rather as a resource to be cultivated. By fostering interprofessional dialogue, healthcare organizations can move toward more inclusive, context-sensitive and ethically grounded approaches to quality improvement.

To support such dialogue, we propose that Table 2 be used as a reflective tool by ICU teams and hospital management. By making visible the distinct logics, outputs and barriers at each organizational level, the table can help surface assumptions, clarify expectations and foster mutual understanding. Structured reflection using this tool may facilitate more integrated approaches to quality improvement – bridging professional and organizational silos and recognizing the value of both measurable outcomes and experiential knowledge.

## Implications for research

This study highlights the value of sociological perspectives in unpacking the complex, context-dependent meanings of quality in healthcare. Our findings suggest that quality work is not only a technical or administrative process but also a site of meaning-making, professional negotiation and organizational identity. As such, there is a pressing need for research that bridges disciplinary boundaries – bringing together social sciences, caring science and medicine – to better understand and support reflexive team development.

We argue that transdisciplinary research, specifically the collaboration between sociologists and clinical actors within healthcare, is not only methodologically fruitful but epistemologically necessary. In an era where healthcare is governed by indicators and quantification, sociology's ability to make the invisible visible – relationships, emotions, ethics and tacit knowledge – is imperative. At the same time, sociology is challenged to develop concepts and methods that can engage in dialogue with other knowledge forms – not from above, but through mutual reflexivity. By engaging in collaborative inquiry, researchers can help develop more inclusive, ethically grounded and practically relevant approaches to quality improvement – approaches that recognize the value of both measurable outcomes and the lived, relational dimensions of care.

## Notes

1. Written consent was obtained to include this statement, which was given outside of the formal interview situation.

2. Quotes have sometimes been carefully edited for clarity and brevity, and in those cases, editing is indicated in the text. Removed words or incomplete sentences are indicated with three dots: … If entire sentences that do not alter the meaning of the quote have been removed, this is indicated with three dots in square brackets: […]. Words in square brackets are inserted either for clarity or for the purpose of protecting identities.
